# Supplementary material for: A UMLS-based spell checker for natural language processing in vaccine safety
Source: BMC Med Inform Decis Mak. 2007 Feb 12;7:3. doi: 10.1186/1472-6947-7-3 (PMC1805499; doi:10.1186/1472-6947-7-3)
Supplement: Additional file 13 — RAP application directory. Sets up directory for RAP (RDF application for PHP) source code [file 1472-6947-7-3-S13.gz › rap/doc/phpdoc/__filesource/fsource_dataset__datasetDatasetDb.php.html]

xml version="1.0" encoding="iso-8859-1"?


File Source for DatasetDb.php


# Source for file DatasetDb.php

Documentation is available at DatasetDb.php

```
1. <?php
2. // ----------------------------------------------------------------------------------
3. // Class: DatasetDb
4. // ----------------------------------------------------------------------------------


8. /**
9. * Persistent implementation of a Dataset in a database.
10. * A RDF dataset is a collection of named RDF graphs.
11. *
12. * <BR><BR>History:<UL>
13. * <LI>05-29-2005                : First version of this class.</LI>
14. *</UL>
15. * @version  V0.9
16. * @author Daniel Westphal (http://www.d-westphal.de)
17. * @author Chris Bizer <chris@bizer.de>
18. *
19. * @package     dataset
20. * @access    public
21. ***/
22. require_once(RDFAPI_INCLUDE_DIR.PACKAGE_DBASE);

24. class DatasetDb extends Dataset
25. {

27. /**
28. * Reference to databse connection
29. *
30. * @var        resource dbConnection
31. * @access    private
32. */
33. var $dbConnection;

35. /**
36. * Reference to the dbStore Object
37. *
38. * @var        $dbStore dbStore
39. * @access    private
40. */
41. var $dbStore;


44. /**
45. * Name of the Dataset
46. *
47. * @var        string
48. * @access    private
49. */
50. var $setName;


53. /**
54. * Constructor
55. * You can supply a Dataset name.
56. *
57. * @param  ADODBConnection
58. * @param  DbStore
59. * @param  string
60. * @access    public
61. */
62. function DatasetDb(&$dbConnection,&$dbStore,$datasetName)
63. {
64. $this->dbConnection=& $dbConnection;
65. $this->dbStore=&$dbStore;
66. $this->setName= $datasetName;
67. $this->_initialize();
68. }

70. /**
71. * Initialize
72. * Read all needed data into the set
73. *
74. *
75. * @access    private
76. */
77. function _initialize()
78. {
79. $recordSet =& $this->dbConnection->execute("SELECT defaultModelUri
80. FROM datasets where datasetName='".$this->setName."'");

82. $this->defaultGraph=& $this->dbStore->getModel($recordSet->fields[0]);
83. }


87. //    === Graph level methods ========================


91. /**
92. * Sets the Dataset name. Return true on success, false otherwise
93. *
94. * @param  string
95. * @access    public
96. */
97. function setDatasetName($datasetName)
98. {
99. if ($this->dbStore->datasetExists($datasetName))
100. return false;

102. $this->dbConnection->execute("UPDATE datasets SET datasetName='".$datasetName."'
103. where datasetName='".$this->setName."'");

105. $this->dbConnection->execute("UPDATE dataset_model SET datasetName='".$datasetName."'
106. where datasetName='".$this->setName."'");
107. $this->setName=$datasetName;
108. return true;
109. }

111. /**
112. * Return the Dataset name.
113. *
114. * @return string
115. * @access    public
116. */
117. function getDatasetName()
118. {
119. return $this->setName;
120. }

122. /**
123. * Adds a NamedGraph to the set.
124. * @param NamedGraphDb
125. */
126. function addNamedGraph(&$graph)
127. {
128. $graphNameURI=$graph->getGraphName();
129. $this->removeNamedGraph($graphNameURI);
130. $this->dbConnection->execute('INSERT INTO dataset_model VALUES("'.$this->setName.'",'.$graph->modelID.',"'.$graphNameURI.'")');
131. }


134. /**
135. * Overwrites the existting default graph.
136. * @param DbModel
137. */
138. function setDefaultGraph(&$graph)
139. {
140. $this->dbConnection->execute('UPDATE datasets SET defaultModelUri ="'.$graph->modelURI.'"  WHERE datasetName ="'.$this->setName.'"');
141. }

143. /**
144. * Returns a reference to the defaultGraph
145. * @return NamedGraphDb
146. */
147. function & getDefaultGraph()
148. {
149. $defaultGraphURI = $this->dbConnection->GetOne("SELECT defaultModelUri FROM datasets WHERE datasetName ='".$this->setName."'");
150. return ($this->dbStore->getNamedGraphDb($defaultGraphURI,'http://rdfapi-php/dataset_defaultGraph_'.$this->setName));
151. }

153. /**
154. * Returns true, if an defaultGraph exists. False otherwise
155. * @return boolean
156. */
157. function hasDefaultGraph()
158. {
159. return true;
160. }

162. /**
163. * Removes a NamedGraph from the set. Nothing happens
164. * if no graph with that name is contained in the set.
165. * @param string
166. */
167. function removeNamedGraph($graphName)
168. {
169. $this->dbConnection->execute('DELETE FROM dataset_model WHERE datasetName="'.$this->setName.'"  AND graphURI ="'.$graphName.'"');
170. }

172. /**
173. * Tells wether the Dataset contains a NamedGraph.
174. * @param  string
175. * @return boolean
176. */
177. function containsNamedGraph($graphName)
178. {
179. $count= $this->dbConnection->GetOne('SELECT count(*) FROM dataset_model WHERE datasetName="'.$this->setName.'"  AND graphURI ="'.$graphName.'"');
180. return ($count>0);
181. }

183. /**
184. * Returns the NamedGraph with a specific name from the Dataset.
185. * Changes to the graph will be reflected in the set.
186. * @param string
187. * @return NamedGraphDb or null
188. */
189. function &getNamedGraph($graphName)
190. {
191. if(!$this->containsNamedGraph($graphName))
192. return null;

194. $modelVars =& $this->dbConnection->execute("SELECT models.modelURI, models.modelID, models.baseURI
195. FROM models, dataset_model
196. WHERE dataset_model.graphURI ='" .$graphName ."' AND dataset_model.modelId= models.modelID");

198. return new NamedGraphDb($this->dbConnection, $modelVars->fields[0],
199. $modelVars->fields[1], $graphName ,$modelVars->fields[2]);
200. }

202. /**
203. * Returns the names of the namedGraphs in this set as strings in an array.
204. * @return Array
205. */
206. function listGraphNames()
207. {
208. $recordSet =& $this->dbConnection->execute("SELECT graphURI FROM dataset_model WHERE datasetName ='".$this->setName."'");

210. $return=array();
211. while (!$recordSet->EOF)
212. {
213. $return[] = $recordSet->fields[0];
214. $recordSet->moveNext();
215. }
216. return $return;
217. }

219. /**
220. * Creates a new NamedGraph and adds it to the set. An existing graph with the same name will be replaced. But the old namedGraph remains in the database.
221. * @param  string
222. * @param  string
223. * @return NamedGraphDb
224. */
225. function &createGraph($graphName,$baseURI = null)
226. {
227. $graph =& $this->dbStore->getNewNamedGraphDb(uniqid('http://rdfapi-php/namedGraph_'),$graphName,$baseURI);
228. $this->addNamedGraph(&$graph);

230. return $graph;
231. }

233. /**
234. * Deletes all NamedGraphs from the set.
235. */
236. function clear()
237. {
238. $this->dbConnection->execute("DELETE FROM dataset_model WHERE datasetName ='".$this->setName."'");
239. }

241. /**
242. * Returns the number of NamedGraphs in the set. Empty graphs are counted.
243. * @return int
244. */
245. function countGraphs()
246. {
247. return ($this->dbConnection->GetOne("SELECT count(*) FROM dataset_model WHERE datasetName ='".$this->setName."'"));
248. }

250. /**
251. * Returns an iterator over all {@link NamedGraph}s in the set.
252. * @return IteratorAllGraphsDb
253. */
254. function &listNamedGraphs()
255. {
256. $recordSet =& $this->dbConnection->execute("SELECT graphURI FROM dataset_model WHERE datasetName ='".$this->setName."'");
257. return (new IteratorAllGraphsDb(&$recordSet,&$this));
258. }

260. /**
261. * Tells wether the set contains any NamedGraphs.
262. * @return boolean
263. */
264. function isEmpty()
265. {
266. return ($this->countGraphs()==0);
267. }

269. /**
270. * Add all named graphs of the other dataset to this dataset
271. * @param Dataset
272. */
273. function addAll($otherDataset)
274. {
275. for($iterator = $otherDataset->listNamedGraphs(); $iterator->valid(); $iterator->next())
276. {
277. $this->addNamedGraph($iterator->current());
278. };

280. if ($otherDataset->hasDefaultGraph())
281. {
282. $this->defaultGraph = $this->defaultGraph->unite($otherDataset->getDefaultGraph());
283. }
284. }

286. //    === Quad level methods ========================


290. /**
291. * Adds a quad to the Dataset. The argument must not contain any
292. * wildcards. If the quad is already present, nothing happens. A new
293. * named graph will automatically be created if necessary.
294. * @param Quad
295. */
296. function addQuad(&$quad)
297. {
298. $graphName=$quad->getGraphName();
299. $graphName=$graphName->getLabel();

301. $graph=& $this->getNamedGraph($graphName);

303. if ($graph===null)
304. $graph=& $this->createGraph($graphName);

306. $statement=$quad->getStatement();
307. $graph->add($statement);
308. }

310. /**
311. * Tells wether the Dataset contains a quad or
312. * quads matching a pattern.
313. *
314. * @param Resource
315. * @param Resource
316. * @param Resource
317. * @param Resource
318. * @return boolean
319. */
320. function containsQuad($graphName,$subject,$predicate,$object)
321. {
322. // static part of the sql statement
323. $sql = "SELECT count(*)
324. FROM statements, dataset_model
325. WHERE datasetName ='".$this->setName."' AND statements.modelID=dataset_model.modelId ";

327. if($graphName!=null)
328. {
329. $sql.= " AND graphURI ='".$graphName->getLabel()."'";
330. }

332. // dynamic part of the sql statement
333. $sql .= DbModel::_createDynSqlPart_SPO($subject, $predicate, $object);

335. return (($this->dbConnection->GetOne($sql))>0);
336. }

338. /**
339. * Deletes a Quad from the RDF dataset.
340. * @param Quad
341. */
342. function removeQuad($quad)
343. {
344. $graphName=$quad->getGraphName();$graphName=$graphName->getLabel();
345. //find namedGraph IDs
346. $graphID = $this->dbConnection->GetOne("SELECT modelId FROM dataset_model WHERE graphURI ='$graphName'");

348. // static part of the sql statement
349. $sql = "DELETE FROM statements WHERE modelID = $graphID";

351. // dynamic part of the sql statement
352. $sql .= DbModel::_createDynSqlPart_SPO($quad->getSubject(), $quad->getPredicate(), $quad->getObject());

354. // execute the query
355. if($graphID)
356. $recordSet =& $this->dbConnection->execute($sql);
357. }

359. /**
360. * Counts the Quads in the RDF dataset. Identical Triples in
361. * different NamedGraphs are counted individually.
362. * @return int
363. */
364. function countQuads()
365. {
366. $sql = "SELECT count(*)
367. FROM statements, dataset_model
368. WHERE datasetName ='".$this->setName."' AND statements.modelID=dataset_model.modelId ";

370. return ((int)$this->dbConnection->GetOne($sql));
371. }

373. /**
374. * Finds Statements that match a quad pattern. The argument may contain
375. * wildcards.
376. * @param Resource or null
377. * @param Resource or null
378. * @param Resource or null
379. * @param Resource or null
380. * @return IteratorFindQuadsDb
381. */
382. function &findInNamedGraphs($graphName,$subject,$predicate,$object)
383. {
384. // static part of the sql statement
385. $sql = "SELECT subject, predicate, object, l_language, l_datatype, subject_is, object_is, dataset_model.graphURI
386. FROM statements, dataset_model
387. WHERE datasetName ='".$this->setName."' AND statements.modelID=dataset_model.modelId ";

389. if($graphName!=null)
390. {
391. $sql.= " AND graphURI ='".$graphName->getLabel()."'";
392. }

394. // dynamic part of the sql statement
395. $sql .= DbModel::_createDynSqlPart_SPO($subject, $predicate, $object);

397. // execute the query
398. $recordSet =& $this->dbConnection->execute($sql);


401. return new IteratorFindQuadsDb(&$recordSet,&$this,true);
402. }

404. /**
405. * Finds Statements that match a pattern in the default Graph. The argument may contain
406. * wildcards.
407. * @param Resource or null
408. * @param Resource or null
409. * @param Resource or null
410. * @return IteratorFindQuadsDb
411. */
412. function &findInDefaultGraph($subject,$predicate,$object)
413. {
414. $defaultGraphID = (int)$this->dbConnection->GetOne("SELECT models.modelID FROM datasets, models WHERE datasets.datasetName ='".$this->setName."' AND datasets.defaultModelUri = models.modelURI");
415. // static part of the sql statement
416. $sql = "SELECT subject, predicate, object, l_language, l_datatype, subject_is, object_is
417. FROM statements
418. WHERE modelID ='$defaultGraphID'";

420. // dynamic part of the sql statement
421. $sql .= DbModel::_createDynSqlPart_SPO($subject, $predicate, $object);

423. // execute the query
424. $recordSet =& $this->dbConnection->execute($sql);

426. return new IteratorFindQuadsDb(&$recordSet,&$this,true);
427. }
428. }
429. ?>
```

Documentation generated on Thu, 7 Jul 2005 13:41:11 +0200 by phpDocumentor 1.3.0RC3
